# Salicylic Acid Alleviates the Adverse Effects of Salt Stress on *Dianthus superbus* (Caryophyllaceae) by Activating Photosynthesis, Protecting Morphological Structure, and Enhancing the Antioxidant System

**DOI:** 10.3389/fpls.2017.00600

**Published:** 2017-04-21

**Authors:** Xiaohua Ma, Jian Zheng, Xule Zhang, Qingdi Hu, Renjuan Qian

**Affiliations:** Institute of Subtropical Crops of Zhejiang ProvinceWenzhou, China

**Keywords:** *D. superbus*, salicylic acid (SA), salt stress, growth, photosynthesis, antioxidants

## Abstract

Salt stress critically affects the physiological processes and morphological structure of plants, resulting in reduced plant growth. Salicylic acid (SA) is an important signal molecule that mitigates the adverse effects of salt stress on plants. Large pink *Dianthus superbus* L. (Caryophyllaceae) usually exhibit salt-tolerant traits under natural conditions. To further clarify the salt-tolerance level of *D*. *superbus* and the regulating mechanism of exogenous SA on the growth of *D. superbus* under different salt stresses, we conducted a pot experiment to examine the biomass, photosynthetic parameters, stomatal structure, chloroplast ultrastructure, reactive oxygen species (ROS) concentrations, and antioxidant activities of *D*. *superbus* young shoots under 0.3, 0.6, and 0.9% NaCl conditions, with and without 0.5 mM SA. *D. superbus* exhibited reduced growth rate, decreased net photosynthetic rate (Pn), increased relative electric conductivity (REC) and malondialdehyde (MDA) contents, and poorly developed stomata and chloroplasts under 0.6 and 0.9% salt stress. However, exogenously SA effectively improved the growth, photosynthesis, antioxidant enzyme activity, and stoma and chloroplast development of *D*. *superbus*. However, when the plants were grown under severe salt stress (0.9% NaCl condition), there was no significant difference in the plant growth and physiological responses between SA-treated and non-SA-treated plants. Therefore, our research suggests that exogenous SA can effectively counteract the adverse effect of moderate salt stress on *D*. *superbus* growth and development.

## Introduction

Under natural conditions, abiotic stresses, especially salt stress, has a triple effect on plant growth. First, it decreases water absorption (osmotic effect). Second, it leads to ion imbalance or turbulence in ion homeostasis and third, it results in plant toxicity (ionic effects; Roussos et al., 2007). This negatively affects growth, the morphological structure, energy transformation, photosynthesis, protein synthesis, and lipid metabolism (Munns, 2005; Parida and Das, 2005). To relieve the adverse effects of salinity, plants have evolved effective resistance responses consisting of non-enzymatic antioxidants (ascorbate and tocopherol) and enzymatic antioxidants, such as catalase (CAT), superoxide dismutase (SOD), and peroxidase (POD). However, plants exhibit a high degree of variability response to salt stress according to the species and the developmental stage. Such as the salt-tolerant species, mangrove, could increase the chlorophyll content, enhance the activities of the antioxidant enzymes (CAT and SOD), and decrease the steady-state rates of transpiration and light-saturated rates of photosynthesis to adapt to the increase of salt concentration (Takemura et al., 2000). While the thylakoid swelling and a decrease in the amount of grana stacking was observed in potato plants under the low salt stress (Fidalgo et al., 2004). And Lutts et al. (1995) was reported that there was an ontogenic evolution of salt resistance and the young seedling stage appeared to be the most sensitive to NaCI during vegetative growth. In addition, exogenously applied compounds can dramatically increase plant growth and development and promote the salt tolerance of plants by enhancing enzymatic antioxidants (Gossett et al., 1994).

Salicylic acid (SA), a plant phenolic that is considered a plant hormone endogenous regulator, can affect a range of physiological and biochemical processes in plants and has significant effects on the resistance to abiotic and biotic stress (Yalpani et al., 1994; Durner and Klessig, 1996; Szalai et al., 2000). Evidence indicates that SA is a endogenous signal molecule for the activation of plant growth and plant defense responses to systemic acquired resistance and pathogen attack local (hypersensitive response; Klessig and Malamy, 1994). It has been shown that SA can markedly improve germination under salt stress, and exogenously applied SA can significantly increase plant growth under both saline and non-saline conditions, particularly at the 500 uM/L level (Kováčik et al., 2009). However, evidence concerning the alleviation of salinity stress by exogenous SA is somewhat controversial. For example, Li et al. (2014) and Arfan et al. (2007) reported that spraying SA could counteract moderate salt stress-induced growth inhibition, whereas no improvement occurred at high concentrations of salt stress. However, Cao et al. (2009) indicated that SA deficiency could protect Arabidopsis against moderate salt stress. Therefore, whether the positive action of SA in the alleviation of salt stress is based on salt concentration and plant species must be further elucidated.

Large pink *Dianthus superbus* L. (Caryophyllaceae) is widely used in ornamental horticulture and housing decoration. This is a loosely tufted perennial, a clonal forb flower with a strong primary root and an ascending or creeping rhizome, whose flowers with relatively long calyx tubes produce considerable nectar and scent (Bittrich, 1993). The genus *Dianthus* L. contains approximately 300 species with a worldwide distribution that is centered in the Mediterranean region. Amongst these species, *Dianthus superbus* L, a threatened species in the whole of Europe, Czech Republic (Rosenthal, 2010; Opdekamp et al., 2012), Latvia and Lithuania (Hooftman et al., 2003), has been shown to have important medicinal value due to its analgesic, sedative, and urinary effects.

Evidence indicates that *D*. *superbus* is a salt-tolerant species that can effectively improve the soil structure of saline-alkali soil (Rosenthal, 2010). Reports on *D. superbus* have focused on its distribution and medical composition analysis (Hiromasa and Noboru, 2006), as well as the cultivation technique (Kinga, 2014). However, few studies have been conducted to understand the effect of SA on the growth and physiology of *D*. *superbus* young shoots under salinity stress. Although *D*. *superbus* is recognized as a salt-resistant variety, the maximum salt level that can be endured by these plants remains unclear. Further, it is not clear whether the exogenous application of SA plays a significant role in ameliorating the influence of salt stress on *D*. *superbus*, thus increasing its salinity resistance.

Therefore, the aims of this study were (1) to determine the maximum salt tolerance of *D. superbus* young shoots, and (2) to determine whether exogenous SA could effectively ameliorate the negative effects of different concentrations of salt stress (under 0.3, 0.6, and 0.9% NaCl conditions) on the growth of *D. superbus*. Through the analysis of biomass, membrane injury, photosynthesis, antioxidative enzyme activity, chloroplast ultrastructure, and stomatal structure, we aimed to determine the internal mechanism of *D*. *superbus* under salt stress and SA treatment. It is anticipated that this information will provide a sound theoretical foundation for the expansion of plantations of this ornamental flower in salinization regions.

## Materials and methods

### Plant materials and growth conditions

The pot experiments under different levels of salt stress were conducted in a controlled environment room at the Institute of Subtropical Crops of Zhejiang Province, Wenzhou, China (120°64′E, 28°00′N) in 2015. In March, 1-year-old healthy and homogenous *D. superbus* young shoots were transferred to plastic pots (15 cm height and 12.5 cm inner-diameter with holes at the bottom, one seedling per pot) that were filled with 2.5 kg of peat (pH 6.11). The peat was imported and had an organic matter content of 3.22%, total N content of 0.282%, total P content of 0.198%, and total K content of 1.663%. Three months later, a completely randomized design with 5 replications per treatment and 5 plants per replication was adopted. Eight treatments consisting of four salt concentrations (0, 0.3, 0.6, and 0.9% NaCl) and two SA concentrations (0 and 0.5 mM) were implemented as follows: T1 (distilled water); T2 (distilled water with 0.5 mM SA); T3 (0.3% NaCl); T4 (0.3% NaCl with 0.5 mM SA); T5 (0.6% NaCl); T6 (0.6% NaCl with 0.5 mM SA); T7 (0.9% NaCl); and T8 (0.9% NaCl with 0.5 mM SA). To avoid osmotic shock, the salt solution was gradually added to the soil through eight steps to achieve the final concentrations of 0.3, 0.6, and 0.9%. The SA was dissolved in ethanol, “Tween-20” (0.1% dilute solution) was added, and double-distilled water was used to obtain 0.5 mM SA. The SA was sprayed on the leaves, both the adaxial and abaxial surfaces, twice daily at 7:00 and 18:00 (4 days before the salt treatment). Analyses were carried out after 45 days, when obvious external differences were observed between the plants subjected to different treatments.

### Plant growth and biomass

At the end of experiment (45 days), one intact plant (above-ground shoot and below-ground root) from each replicate per treatment was harvested for biomass determination. The leaf area (LA) (cm^2^) of the third and fourth fully expanded leaves from the top of the shoots was measured at the same time. The fresh weight and biomass, including roots, stems, and leaves, was obtained by electronic scales. And the leaf mass ratio (LMR) was calculated for each individual by dividing the leaf dry mass by total biomass. The specific leaf weight (SLW) was calculated by dividing the fresh leaf weight by the corresponding leaf area (LA) (Tang et al., 2015).

$$\begin{array}{l} \text{Leaf mass ratio }(\text{LMR})\;(\%)\text{​ }=\text{ ​}(\text{Leaf dry mass/total dry mass})\\ \;\times 100\%\\ \text{Specific leaf weight }(\text{SLW})=\text{leaf fresh weight/LA} \end{array}$$

### Photosynthetic parameters

The youngest, fully developed, healthy leaves were randomly selected from the top of the shoots for photosynthetic measurements, using an LI-6400 XT portable photosynthesis system (Li-Cor, Inc., Lincoln, NE, U.S.) with a standard leaf chamber equipped with a 6400-02B LED light source. Measurements were conducted on sunny day from 8:30 to 11:30; the leaf temperature was maintained at 28 ± 2°C, the CO_2_ concentration ([CO_2_]) in the leaf chamber was 400 ppm, relative humidity (RH) was 50%, and the photosynthetic photon flux density was 1,200 μmol m^−2^ s^−1^. LA was analyzed at the same time and used when calculating the photosynthetic parameters (Ma et al., 2015; Tang et al., 2015).

### Photosynthetic pigments

Approximately 0.1 g of finely cut and well-mixed leaf tissues were repeatedly extracted using 8 mL of 95% alcohol. Chlorophyll was extracted for 24 h in the dark, and the samples were shaken three times until blanched. The absorbance of the supernatant was measured at 649, 665, and 470 nm using a spectrophotometer (Shimadzu UV-2550, Kyoto, Japan) after centrifugation of the mixture. Chlorophyll concentrations were calculated using a standard method (Lichtenthaler, 1987) and expressed as mg g^−1^ fresh weight (FW).

### Determination of the proline content, lipid peroxidation, and membrane permeability

The proline content was expressed as relative electrolyte leakage rate and determined by the method of Shen et al. (2014). The proline concentration was calculated using proline standards (0–50 mg ml^−1^) in an identical manner (Shen et al., 2014).

The lipid peroxidation level was assessed based on the malondialdehyde (MDA) content using a method described by Guidi et al. (2000). The MDA concentration was expressed as mol g^−1^ using the following formula: 6.45(A532–A600)–0.56A450.

Membrane permeability was assessed by measuring the relative electrolyte conductivity of the leaf following the method described by Deshmukh et al. (1991). Leaves (0.2 g) were rinsed using distilled water and immersed in a test tube with 30 mL of distilled water for 12 h. The electrical conductivity (EC1) of the solution was measured using a conductivity meter (Model DJS-1C; Shanghai Analytical Instrument Co. Shanghai, China). Then, the samples were heated at 100°C for 30 min and the conductivity (EC2) in the bathing solution was determined. Membrane permeability was calculated as the ratio of EC1/EC2.

### Determination of the superoxide anion (${\text{O}}_{2}^{.-}$) production rate and the peroxide (H_2_O_2_) content

The ${\text{O}}_{2}^{.-}$ production rate was measured by monitoring the nitrite formation from hydroxylamine in the presence of ${\text{O}}_{2}^{.-}$ as described by Wang and Luo (1990) with a slight modification. The ${\text{O}}_{2}^{.-}$ production rate was expressed as μM min^−1^ g^−1^ protein.

For analysis of the H_2_O_2_ content, 0.2 g of leaf tissue was ground finely and homogenized with 25 mL of acetone at 0°C using the method described by Patterson et al. (1984). The H_2_O_2_ content was calculated using H_2_O_2_ as a standard and expressed as mM g^−1^ protein.

### Determination of superoxide dismutase (SOD), catalase (CAT), and peroxidase (POD) activity

To obtain a crude enzyme extract, 0.3 g of frozen leaves were ground in a mortar with 8 mL of 50 mM phosphate buffer solution (pH 7.8) containing 1% polyethylene pyrrole (PVP) at 4°C. The homogenate was centrifuged at 10,000 rpm for 15 min at 4°C. The supernatant was collected to measure the enzyme activities.

SOD activity was analyzed by monitoring its ability to inhibit the photochemical reduction of nitroblue tetrazolium (Giannopotitis and Ries, 1977). One unit of SOD activity was defined as the amount of enzyme that resulted in 50% inhibition of reduction of nitroblue tetrazolium. The specific SOD activity was expressed as U g^−1^ FW protein. CAT activity was measured by monitoring the disappearance of H_2_O_2_ (Díaz-Vivancos et al., 2008). One CAT unit was defined as the amount of enzyme needed to decompose 1 mmol H_2_O_2_ min^−1^ under these assay conditions. The specific CAT activity was expressed as U g^−1^ FW min^−1^. The POD activity in the leaves was estimated using guaiacol as the substrate, following Thomas et al. (1982). Specific POD activity is expressed as U g^−1^ FW min^−1^.

### Analysis of stomatal structure and chloroplast ultrastructure

Fully expanded leaves from the same leaf position were harvested from all treatment seedlings under daylight conditions. Square samples were cut from the middle of the leaf and fixed with 2.5% glutaraldehyde in phosphate buffer (0.1 M pH7.0) for more than 4 h, then washed three times in the phosphate buffer (0.1 M pH7.0) for 15 min at each step, next postfixed with 1% OsO_4_ in phosphate buffer for 1–2 h and washed three times in the phosphate buffer (0.1 M pH7.0) for 15 min at each step. Then the sample was first dehydrated by a graded series of ethanol (30, 50, 70, 80, 90, 95, and 100%) for about 15–20 min at each step, transferred to the mixture of alcohol and iso-amyl acetate (v:v = 1:1) for about 30 min, then transferred to pure iso-amyl acetate for about 1 h or overnight. In the end, the sample was dehydrated in Hitachi Model HCP-2 critical point dryer with liquid CO_2_. The dehydrated sample was coated with gold-palladium in Hitachi Model E-1010 ion sputter for 4–5 min and observed in Hitachi Model TM-1000 SEM (Hitachi, Japan). For morphometric estimation of stomatal aperture and density, the methods described by Snider et al. (2009) were used.

To examine the chloroplast ultrastructure of mesophyll cells, leaf samples were immediately fixed in 2.5% (v/v) glutaraldehyde (0.1 M phosphate buffer, pH 7.2) for at least 4 h after being cut from the plants. Then, the samples were immersed in 1% (v/v) osmium acid for post-fixation 1–2 h and washed three times in the phosphate buffer (0.1 M, pH7.0) for 15 min at each step. The specimen was first dehydrated by a graded series of ethanol (30, 50, 70, 80, 90, 95, and 100%) for about 15–20 min at each step, then transferred to absolute acetone for 20 min. Then samples were placed in 1:1 mixture of absolute acetone and the final Spurr resin mixture for 1 h at room temperature, then transferred to 1:3 mixture of absolute acetone and the final resin mixture for 3 h and to final Spurr resin mixture for overnight. And then specimen was placed in eppendorf contained Spurr resin and heated at 70°C for more than 9 h. At the end, the specimen was sectioned in LEICA EM UC7 ultratome and sections were stained by uranyl acetate and alkaline lead citrate for 5–10 min respectively and observed in Hitachi Model H-7650 TEM (H7650, Hitachi, Tokyo, Japan; Deng et al., 2012). The methods of microscopic measurement was described by Ma et al. (2015).

### Data analysis

Data were tested using analysis of variance (ANOVA) with SPSS software version 16.0 (SPSS, Chicago, IL, U.S.), Duncan's multiple range test was used to detect differences between means. The *P*-value was set at 0.05 and 0.01 for the ANOVA and Duncan's multiple range tests, respectively.

## Results

### Plant growth and development of *D. superbus* under different salt stresses and SA treatments

**Figure 1:**
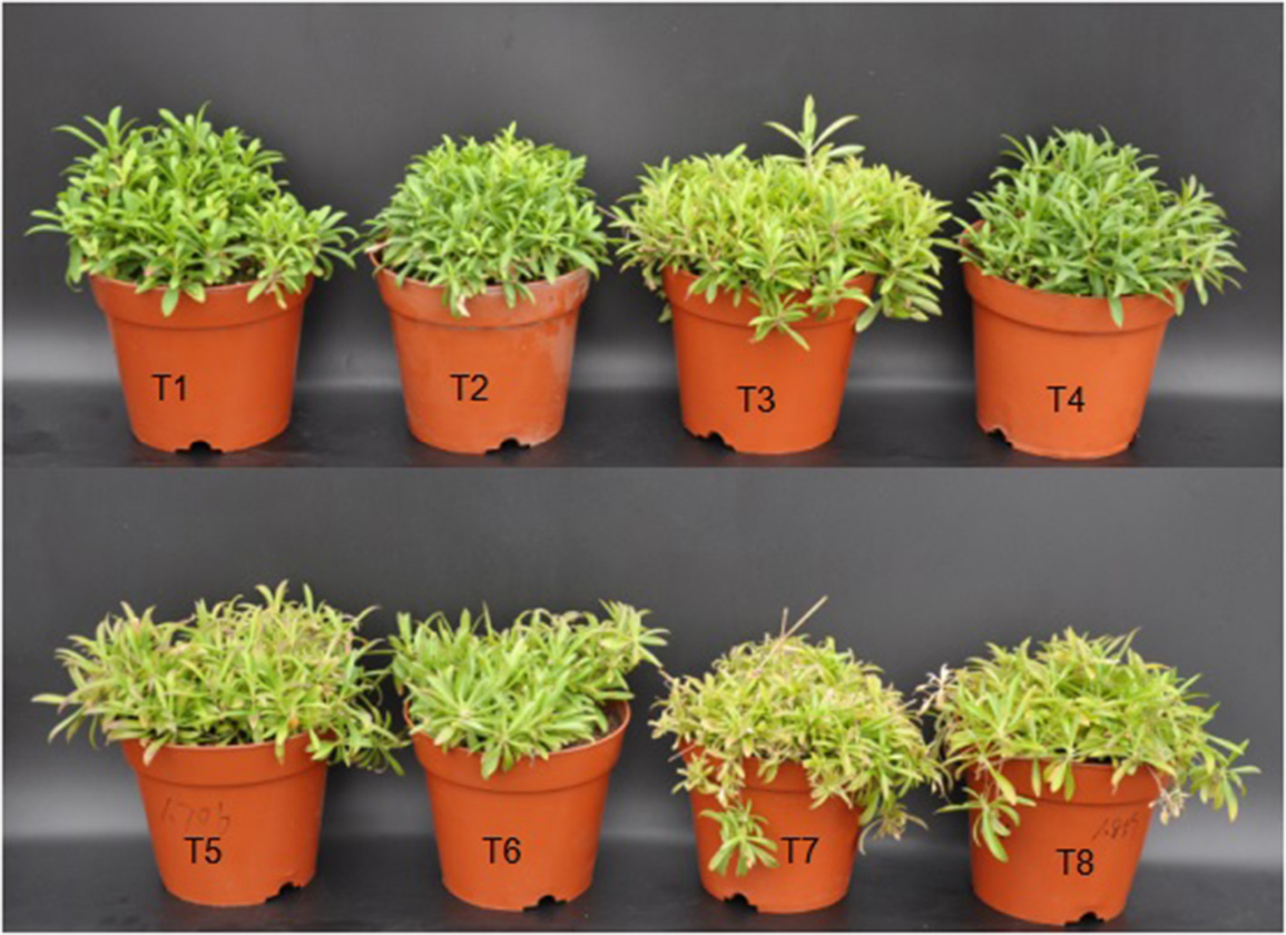
**Effects of SA on the growth in *D. superbus* grown under salt stress (means ± *SD*)**. T1, distilled water; T2, distilled water with 0.5 mmol SA; T3, 0.3% NaCl; T4, 0.3% NaCl with 0.5 mmol SA; T5, 0.6% NaCl; T6, 0.6% NaCl with 0.5 mmol SA; T7, 0.9% NaCl; T8, 0.9% NaCl with 0.5 mmol SA.

Salinity treatments significantly suppressed plant growth and development, resulting in a pronounced reduction in the fresh weight and dry biomass of *D. superbus*. Compared with the non-salt-treated *D. superbus* (T1), the 0.3, 0.6, and 0.9% NaCl treatments markedly reduced the fresh weight of the seedlings by 10.7, 28.13, and 38.5%, respectively (Table S1, Figure 1). The SA treatment reduced the decrease in the growth of the salt-stressed *D. superbus*, and there was no significant difference between non-salt treatment (T1) and the non-salt treatment with SA (T2). Compared with the salt-stressed plants without SA treatment under 0.3%, 0.6% NaCl conditions, the fresh weight and biomass increased by 17.23 and 8.27% under 0.3% NaCl with SA, and by 34.01 and 12.86%, respectively, under 0.6% NaCl with SA. However, there was no significant difference in the fresh weight between the treatments with and without SA under 0.9% NaCl stress. Whereas, SA increased the biomass allocated to the leaves of *D. superbus* under 0.6 and 0.9% salt-treated (Table S1). Interestingly, the specific leaf weight (SLW) did not change significantly with 0.3% NaCl (*P* > 0.05), compared with the non-salt-treated *D. superbus* (T1) but was markedly increased with 0.6 and 0.9% NaCl (*P* < 0.05).

### Photosynthetic parameters of *D. superbus* under different salt stress and SA treatments

Salt stress inhibited photosynthesis, with decreases in the net assimilation rate (Pn), transpiration rate (Tr), and stomatal conductance (Gs) of the leaves of 10.2, 12.2, and 22.6% under the 0.3% NaCl condition, 23.1, 20.9, and 32.2% under the 0.6% NaCl condition, and 30.1, 25.8, and 32.2%, respectively, under the 0.9% NaCl condition (Table S2). Notably, the intercellular CO_2_ (Ci) under 0.9% NaCl was significantly higher than the other treatments (*P* < 0.05). The SA treatment increased the Pn, Tr, Ci, and Gs of leaves under 0.3 and 0.6% salt stress. The Pn and Tr of SA-treated plants increased by approximately 9.02 and 10.2% under the 0.3% NaCl condition and 8.7 and 4.8%, respectively, under the 0.6% NaCl condition compared with the non-SA-treated plants. The Ci in the SA-treated plants under the 0.9% NaCl condition was similar to that in the non SA-treated plants (*P* > 0.05). However, the Ci in the SA-treated plants under 0.3 and 0.6% NaCl stress was markedly higher (8.7 and 3.5%, respectively) than that of the plants without SA treatment (*P* < 0.05). Furthermore, there were no obvious differences in the Pn-, Gs-, Tr-, and Ci-values between the SA treatment (T2) and the water-treated (without SA) plants (T1) and between the SA (T8) and 0.9% NaCl treatment without SA (T7) (*P* > 0.05).

### Photosynthetic pigment content of *D. superbus* under different salt stress and SA treatments

**Figure 2:**
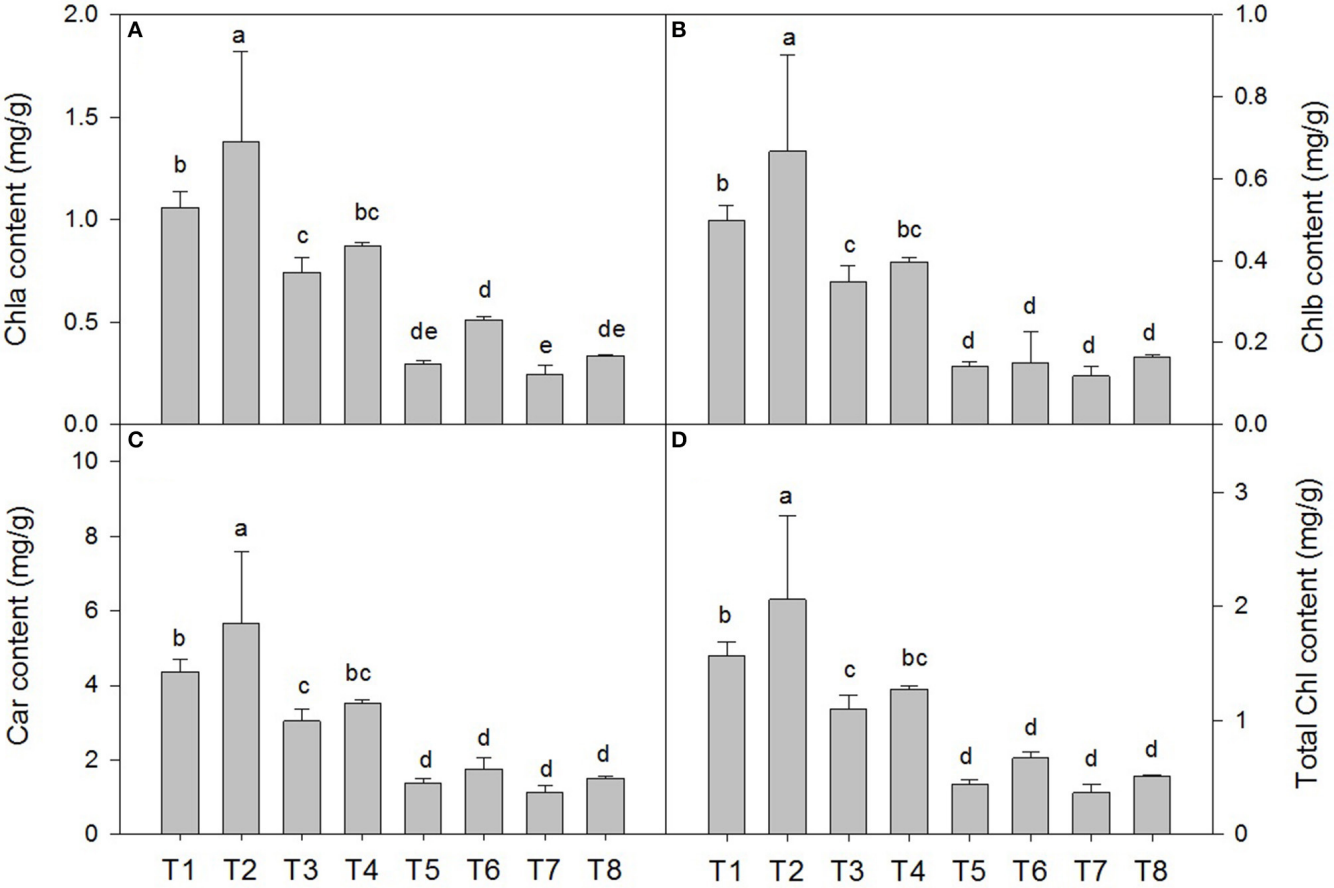
**Effects of SA on the chlorophyll a (chla) (A)**, chlorophyll b (chlb) **(B)**, carotenoid (car) **(C)** and total chlorophyll content **(D)** in *D. superbus* grown under salt stress (means ± *SD*). Different letters indicate significant differences (*P* < 0.05) according to an LSD-test; the same letter indicates no significant differences between the treatments, *n* = 5.

Salt stress resulted in a decrease in the chla, chlb, total chlorophyll and carotenoid contents compared with those of the non-salt-treated plants. The chla content decreased by 29.5% under the 0.3% salt condition, 72.3% under the 0.6% salt condition, and 77.1% under the 0.9% saline condition (Figure 2A). The SA treatment under salt stress conditions slowed the decrease in the chla content by approximately 16.2% under the 0.3% saline condition and 41.3% under the 0.6% saline condition compared with the non-SA-treated plants (Figure 2A). The chlb content decreased by 20.4% under the 0.3% salt condition, 71.4% under the 0.6% saline condition, and 77.5% under the 0.9% saline condition (Figure 2B). Under salt stress conditions, the SA treatment slowed the decrease in the chlb content by approximately 14.7% under the 0.3% saline condition and by 7.1% under the 0.6% saline condition compared with the non-SA-treated plants (Figure 2B). Similar responses were observed in the carotenoid content (Figure 2C). However, there were no significant differences in the chla, chlb, and carotenoid contents between the treatments with and without SA under 0.9% NaCl stress.

### Malondialdehyde (MDA) content, relative electric conductivity (REC), and proline content of *D*. *superbus* seedlings under different salt stress and SA treatments

**Figure 3:**
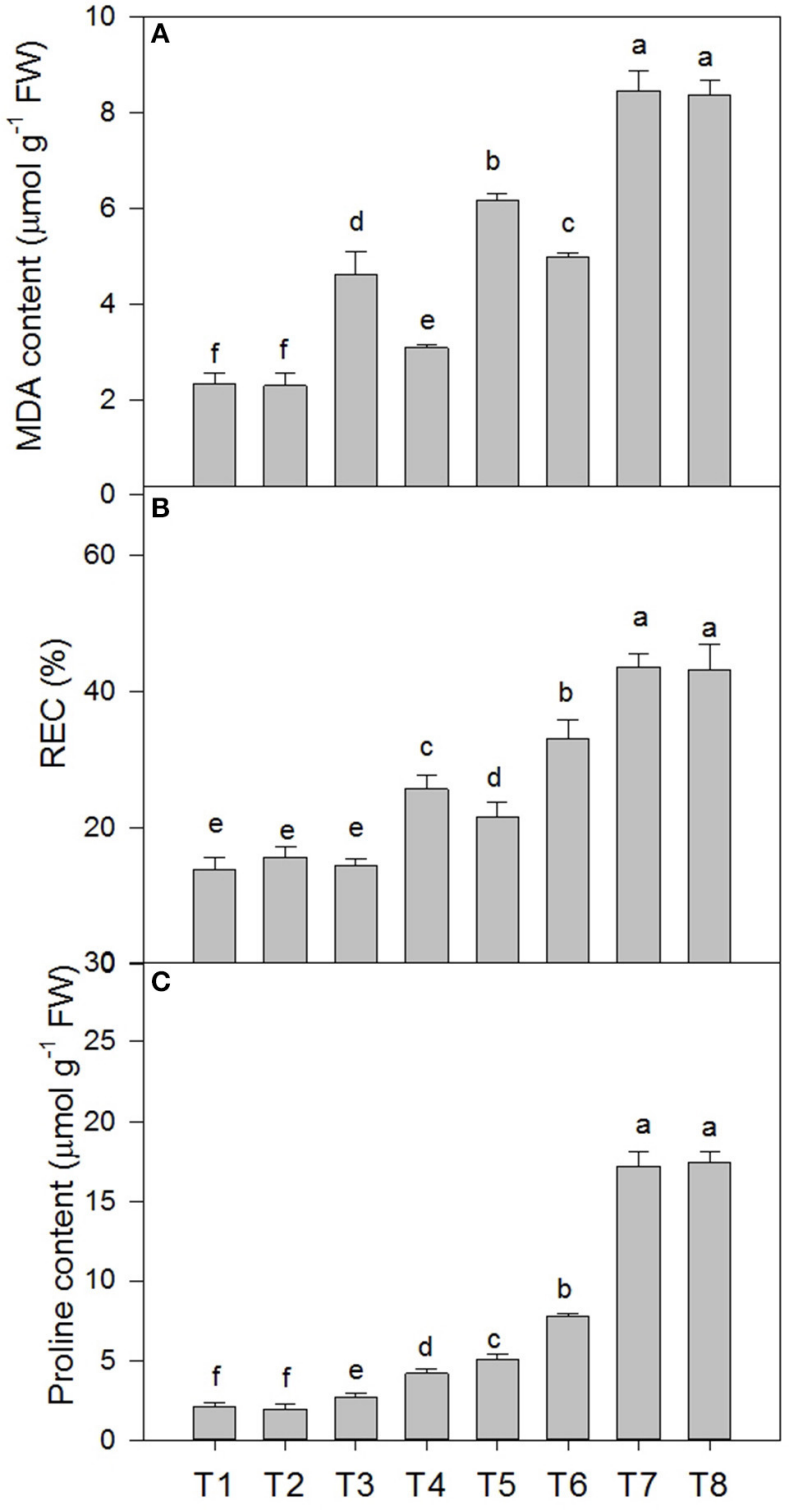
**Effects of SA on the malondialdehyde (MDA) content (A)**, relative electric conductivity (REC) **(B)**, and proline content **(C)** of *D*. *superbus* grown under salt stress (means ± SD). Different letters indicate significant differences (*P* < 0.05) according to an LSD-test; the same letter indicates no significant differences between the treatments, *n* = 5.

Salt stress caused membrane lipid peroxidation injury in the leaves of the *D*. *superbus* (Figure 3). The MDA content increased by 98.2, 162.1, and 264.2% under the 0.3, 0.6, and 0.9% NaCl conditions, respectively, and the relative electrical conductivity (REC) increased by 4.38, 55.1, and 139.1% under the 0.3, 0.6, and 0.9% NaCl conditions, respectively. In addition, the proline content increased by 28.6, 139.8, and 714.6% under the 0.3, 0.6, and 0.9% NaCl conditions, respectively. Compared with the non-SA-treated plants, the reductions in MDA in the SA-treated plants were approximately 33.0% (*P* < 0.05) under the 0.3% NaCl condition and 18.9% (*P* < 0.05) under the 0.6% NaCl condition. However, compared with the non-SA-treated plants, the REC and proline content increased by 76.8 and 53.7% under 0.3% salt stress, and by 52.8 and 54.1%, respectively, under 0.6% salt stress. Interestingly, no significant difference was found between MDA, REC and the proline content between the SA (T2) and water-treated (without SA) plants (T1) (*P* > 0.05) and between the SA-treated plants with 0.9% NaCl (T8) and those without SA that were subjected to 0.9% NaCl (T7) (*P* > 0.05).

### Determination of the superoxide anion (${\text{O}}_{2}^{.-}$) production rate and the peroxide (H_2_O_2_) content of *D. superbus* under different salt stress and SA treatments

**Figure 4:**
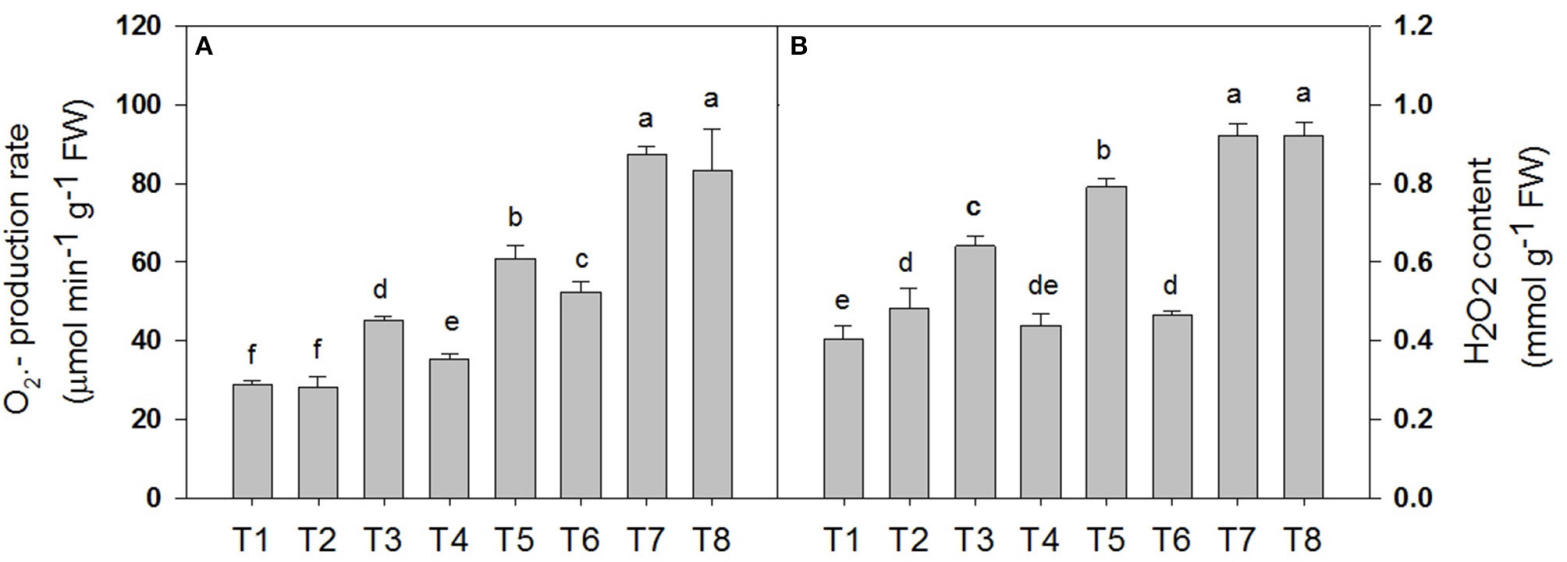
**Effects of SA on the superoxide anion (${\text{O}}_{2}^{.-}$) production rate (A)** and the peroxide (H_2_O_2_) content **(B)** of *D. superbus* grown under salt stress (means ± *SD*). Different letters indicate significant differences (*P* < 0.05) according to an LSD-test; the same letter indicates no significant differences between the treatments, *n* = 5.

Salt stress accelerated the production of ROS in the leaf tissue of the *D*. *Superbus* (Figure 4). The ${\text{O}}_{2}^{.-}$ production rate was significantly increased by 56.5% (*P* < 0.05), 111.4% (*P* < 0.05), and 203.1% (*P* < 0.05) under the 0.3, 0.6, and 0.9% NaCl conditions, respectively, and the H_2_O_2_ content increased by 60.0% (*P* < 0.05), 97.5% (*P* < 0.05) and 130.0% (*P* < 0.05) under the 0.3, 0.6, and 0.9% NaCl conditions, respectively. Compared with the non-SA-treated plants, the reductions in the ${\text{O}}_{2}^{.-}$ production rate and the H_2_O_2_ content in the SA-treated seedlings were approximately 21.5% (*P* < 0.05) and 31.2% (*P* < 0.05) under the 0.3% NaCl condition and 18.9% (*P* < 0.05), 14.1% (*P* < 0.05) (*P* < 0.05) under the 0.6% NaCl condition, respectively. In addition, no significant difference was found in the ${\text{O}}_{2}^{.-}$ production rate and the H_2_O_2_ content between the SA (T2) and water-treated (without SA) plants (T1) (*P* > 0.05), and between the SA plants (T8) and the 0.9% NaCl without SA plants (T7) (*P* > 0.05).

### Superoxide dismutase (SOD), catalase (CAT), and peroxidase (POD) activity of *D. superbus* under different salt stress and SA treatments

**Figure 5:**
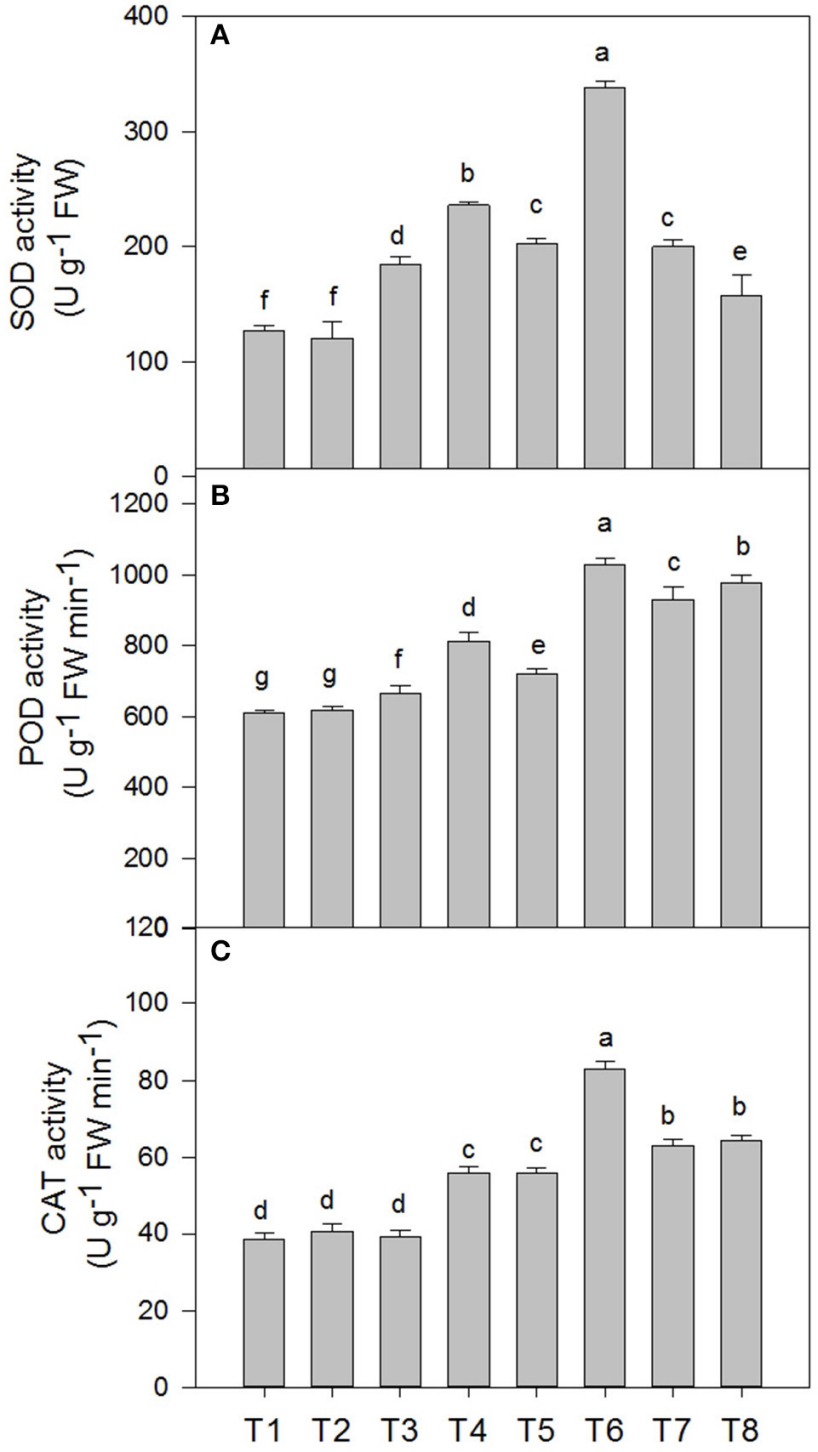
**Effects of SA on the superoxide dismutase (SOD) (A)**, peroxidase (POD) **(B)**, and catalase (CAT) **(C)** activities of *D*. *superbus* grown under salt stress (means ± *SD*). Different letters indicate significant differences (*P* < 0.05) according to an LSD-test; the same letter indicates no significant differences between the treatments, *n* = 5.

The superoxide dismutase (SOD), peroxidase (POD) and catalase (CAT) activities in the *D*. *superbus* were significantly affected by salt tress and SA treatment (Figure 5). SOD increased by 46.3% (*P* < 0.05), 60.3% (*P* < 0.05), and 58.3% (*P* < 0.05) under the 0.3, 0.6, and 0.9% NaCl conditions, respectively. In addition, POD increased by 8.6, 18.1% (*P* < 0.05) and 52.1% (*P* < 0.05) under the 0.3, 0.6, and 0.9% NaCl conditions, respectively. However, CAT increased by 44.2% (*P* < 0.05) and 63.4% (*P* < 0.05) under the 0.6 and 0.9% NaCl conditions, respectively, but no significant difference was found between the non-salt treatment (T1) and the 0.3% NaCl condition (T3). The SA treatment enhanced the superoxide dismutase (SOD), peroxidase (POD) and catalase (CAT) activities compared with the non-SA-treated plants under salt stress. SOD increased by 27.7% (*P* < 0.05) and 67.18% (*P* < 0.05) under the 0.3% NaCl conditions and 0.6% NaCl conditions, respectively, compared with the non-SA-treated plants. The peroxidase (POD) and catalase (CAT) activities increased by 22.4%(*P* < 0.05), 42.6% (*P* < 0.05), and 42.1% (*P* < 0.05), 48.8% (*P* < 0.05) under the respective 0.3 and 0.6% NaCl conditions, compared with the non-SA-treated plants. However, there was no significant difference in the superoxide dismutase (SOD), peroxidase (POD), and catalase (CAT) activities between the SA (T2) and water-treated (without SA) plants (T1) (*P* > 0.05).

### Stomatal structure of *D*. *superbus* under different salt stress and SA treatments

**Figure 6:**
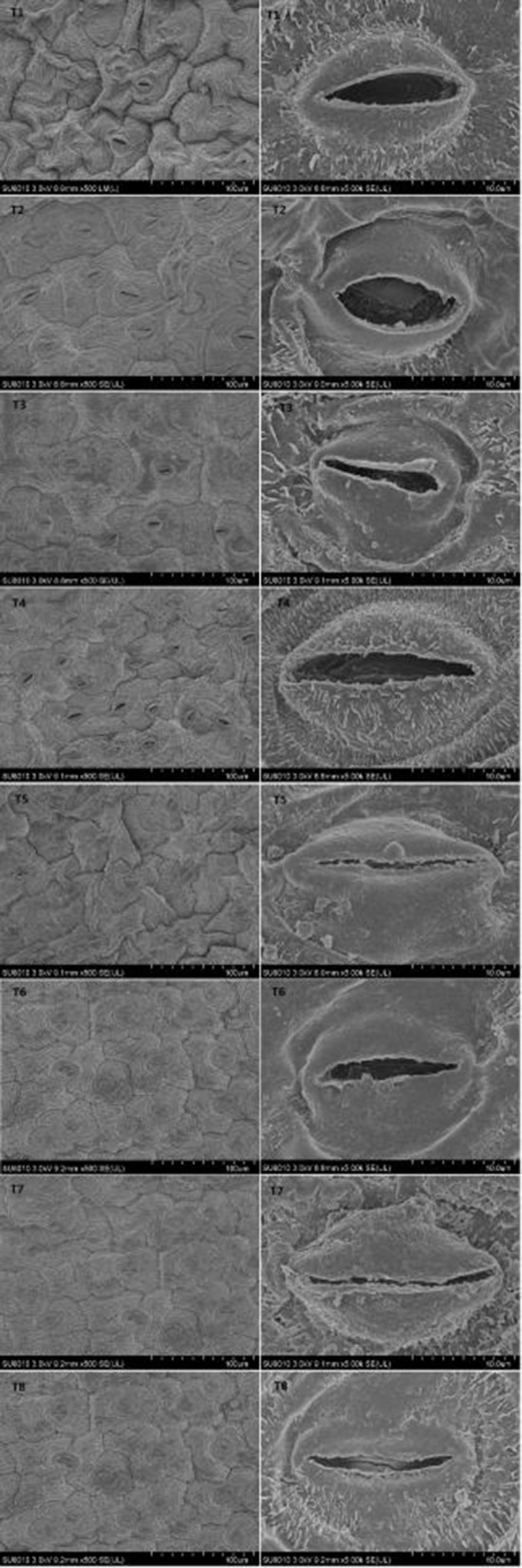
**Effects of SA on stomatal structure of *D*. *superbus* grown under salt stress (means ± *SD*)**.

Salt stress resulted in a variation in the stomatal density and aperture compared with the non-salt-treated plants. Different salt stress treatments were associated with differences in the stomatal traits (Figure 6, Table S3). When salt stress increased from 0 to 0.9%, a gradual decrease in the stomatal aperture was observed. However, the SA treatment under salt stress conditions slowed the decrease in stomatal aperture under different salt stress conditions. And the stomata density decreased from the 0% to the 0.9% salinity treatment. While the SA treatment under 0.3 and 0.6% salt stress increased stomata density.

### Chloroplast ultrastructure of *D. superbus* under different salt stress and SA treatments

**Figure 7:**
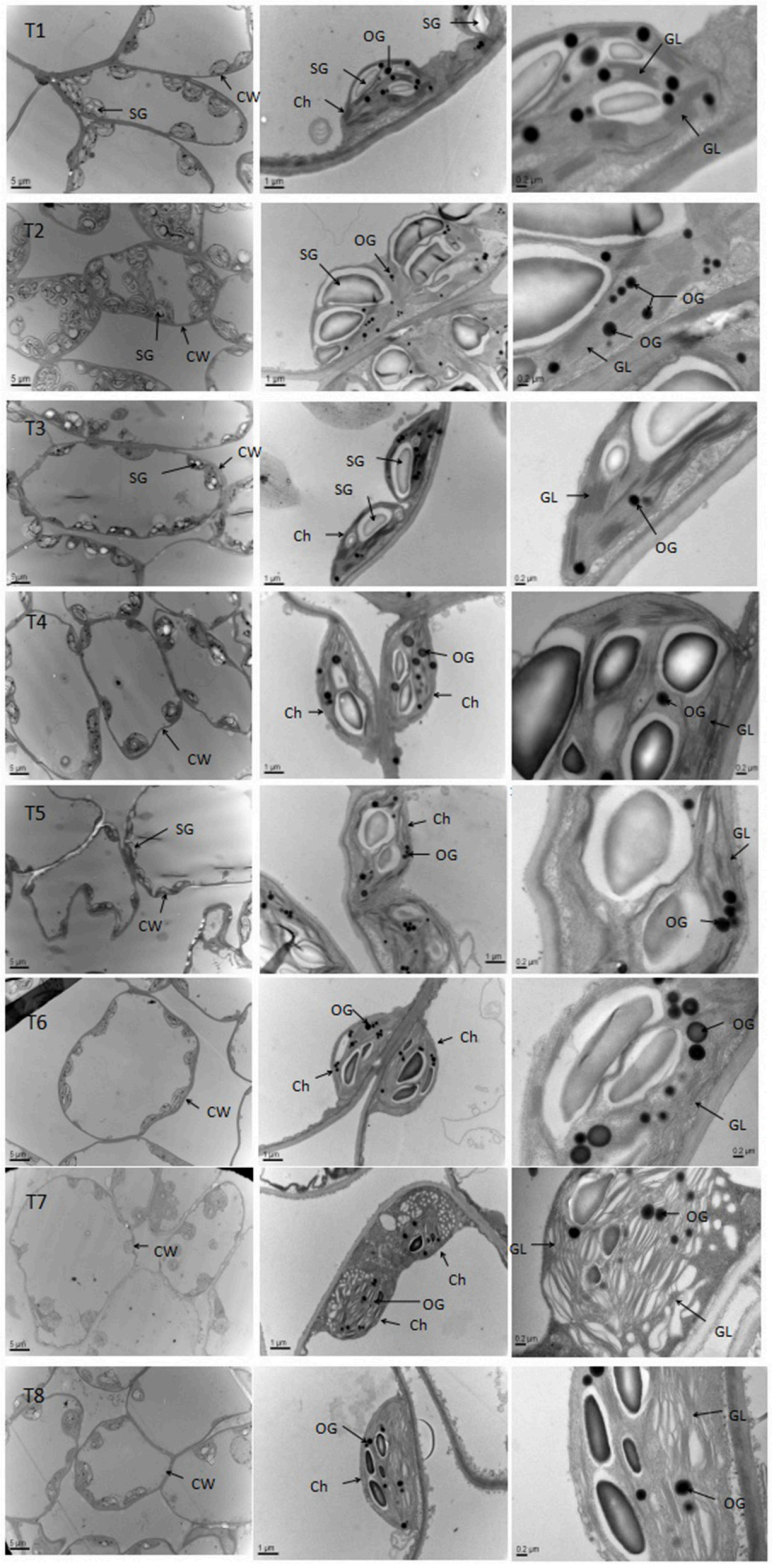
**Effects of SA on the chloroplast ultrastructure of *D*.* superbus* grown under salt stress (means ± *SD*)**. Ch, chloroplast; CW, cell wall; SG, starch grains; OG, osmiophilic granule; GL, grana lamella.

Salt stress caused obvious changes in the shape, size of chloroplasts (Figure 7, Table S4). The numbers of grana decreased with increasing salt treatment, and the shape of the chloroplasts varied from ellipsoid to swollen oblate or spheroidal. The chloroplasts in the leaves of seedlings grown under the non-salt treatment (T1) and non-salt treatment with SA (T2) showed a normal ultrastructure, with typical grana, well-organized stroma thylakoids and a maximum number of grana lamella. However, the stroma thylakoids became more irregular and disordered, with teas and faults, and the grana lamella decreased with salt stress from 0 to 0.9%. The plants grown under SA treatment mostly had grana containing more and well-organized thylakoids than those plants grown under salt stress. In particular, the number of osmiophilic granules increased with salt stress from 0 to 0.9%, whereas the number of osmiophilic granules decreased under SA treatment compared with those plants grown under salt stress. Overall, the chloroplasts of the non-salt-treated seedlings (T1) and the non-salt-treated seedlings with SA (T2) developed the best.

## Discussion

Salt stress restricts plant growth and morphology by adversely influencing various aspects of physiology and biochemistry, such as photosynthesis, superoxide ion homeostasis, antioxidant responses, osmolyte accumulation, and proline metabolism (Misra and Saxena, 2009; Roussos et al., 2013). Generally, SA is used in plant cultivation due to its ability to regulate the plant resistance response to different environmental stresses, particularly salt stress (Senaratna et al., 2000; Hayat et al., 2010). Our results clearly suggested that *D*. *superbus* is a salt-tolerant plant, and exogenous supply of SA could alleviate the deleterious effects of moderate salinity on the growth of *D. superbus*, through enhanced activation of the photosynthetic process and the relief of membrane injury.

*D. superbus* under 0.3% salt stress showed no apparent inadaptability due to the saline conditions. Furthermore, to a certain extent, the exogenous SA counteracted the salt stress-induced growth inhibition of *D. superbus* under 0.6% salinity, but no improvement occurred under 0.9% salinity. In the study, salt stress markedly reduced the fresh weight by 28.13 and 41.37% after treatment with 0.6 and 0.9% NaCl, respectively, for 45 days (Table S1). The observed significant decreases in fresh weight and biomass suggested that salinity inhibited growth and caused damage to the *D. superbus*. These results provide further evidence that the *D. superbus* are more tolerant of salt stress than general herbaceous plants. Furthermore, increasing evidence suggests that SA treatment significantly alleviates the deleterious effects of salinity on plant growth (Shakirova et al., 2003). Iqbal et al. (2006) showed that SA supply improved the adverse effects of salt stress on the growth of wheat cultivars. It has also been reported that SA-treated maize plants had higher dry mass compared with untreated plants that were also grown under salt stress (Gunes et al., 2007). The variation in allocation of biomass to different organs may be crucial to the success of a seedling to adapting to a new environment (Tang et al., 2015). Higher biomass allocation to leaves occurred with an increase in the SA-treated *D. superbus* under 0.6 and 0.9% salt stress (Table S1). This may be a common response among herbaceous species in several ecotope (Maliakal et al., 1999). According to the results of this study, we conclude that SA significantly modulated the salt stress response of *D. superbus* and further suggest that SA can act as a potential regulator to improve plant growth under salt stress.

Some earlier reports showed that SA-induced increases in growth could be due to the SA-enhanced increase in the net photosynthetic rate under salt stress, particularly at the 0.5 mM SA level (Li et al., 2014). Our study also found that salinity stress significantly affected the photosynthesis processes, especially the net photosynthetic rate (Pn), which was similar to Li et al. (2014) reported in *Torreya grandis*. The Pn decreased by 10.2% (*P* < 0.05), 32.2% (*P* < 0.05), and 30.1% (*P* < 0.05) from 0.3 to 0.9% salt compared with the non NaCl-treated plants (Table S2). Photosynthesis is a critical metabolic pathway in plants. The maintenance of a favorable photosynthetic rate implies the maintenance of growth under salt stress. Studies have shown that SA can efficiently ameliorate photosynthetic damage in *Arabidopsis* (Borsani et al., 2001), tomato (Molina et al., 2002), and cucumber seedlings (Shim et al., 2009). In this study, the Pn of *D. superbus* with the SA supplement increased by approximately 9.02 and 4.8% under the 0.3 and 0.6% NaCl conditions, respectively, compared with the non-SA-treated plants (Table S2). The observed changes in the photosynthetic rate could be considered due to stomatal and/or non-stomatal limitations (Dubey, 2005). Salt stress caused an indirect effect, i.e., stomatal closure, which caused a reduction in the CO_2_ supply, thereby resulting in the decline in the photosynthetic rate. Under the 0.6% NaCl condition, both the Pn and stomatal conductance (Gs) decreased compared to the non-salt-treated *D. superbus*, demonstrating that the decrease in the photosynthetic rate was a result of stomatal closure. The SA treatment relieved the adverse effects of salinity stress on photosynthesis by adjusting Pn and Gs. However, non-stomatal limitations, such as the membrane structural integrity of the photosynthetic apparatus, the energy supply of the photosynthetic reaction center, and the associated enzyme activities, also affected the Pn under salt stress (Li et al., 2014). Our study indicated that the Pn decreased continuously in the 0.9% NaCl treatment, while the similar changes in Gs also existed but not with similar response of Pn. These results suggest that either non-stomatal limitations or both stomatal and non-stomatal factors may have impacted the photosynthetic process under the 0.9% salt condition. Furthermore, the SA treatment did not have an obvious effect (i.e., an improvement) on photosynthesis under the 0.9% salt condition, which indicated that the exogenous SA was not efficient in improving the photosynthetic process to counteract the stress damage under the 0.9% salt treatment.

Studies have shown that the photosynthetic pigment content is one of the critical factors determining photosynthetic efficiency and plant growth (Shao et al., 2014). In this study, salt stress obviously decreased the Chla and Chlb contents, whereas the SA treatment diminished this reduction of the chlorophyll contents of *D. superbus* under salt stress (Figure 2). These results are consistent with studies on *T. grandis*, wheat and barley (Li et al., 2014). SA might promote the activity of enzymes related to chlorophyll biosynthesis or might relieve the impairment of the photosynthetic system, thereby reducing chlorophyll degradation. Therefore, these conclusions suggest that the increased chlorophyll content in the plants supplied with SA may be an important means to improve photosynthesis and further relieve the salt stress damage in *D. superbus*.

Studies usually consider the malonaldehyde (MDA) content as an important indicator to reflect the level of lipid peroxidation (Shen et al., 2014). In our study, salt stress significantly enhanced the MDA content in *D. superbus* from the 0.3% to the 0.9% treatments, which indicated that salinity caused a high level of oxidative damage in the lipid membranes of these seedlings. However, SA increased the activity of SOD, CAT, and POD, thus reducing ROS and, as a result, oxidative damage to membranes. These results are similar to those presented by Shen et al, who conducted a study on *T. grandis*, suggesting that SA mediates the peroxidatic reaction, which leads to membrane injury in the oxidative response, and acts as a natural signal molecule for the activation of plant defense responses (Klessig and Malamy, 1994). Evidence indicates that relative electrolytSic leakage (REC) is a direct indicator reflecting the level of membrane permeability. Our results clearly indicated that RWC increased in response to salt stress, and compared with the non-SA-treated plants, SA-treated *D. superbus* under salt stress showed a small increase in RWC. This is consistent with Li et al. (2014), who found that exogenous SA treatment could increase the growth of *T. grandis* seedlings by promoting the RWC of the leaf. The proline content is usually considered to be related to stress resistance mechanisms, and this is one of the most frequently reported modifications induced by salt stress in plants (Misra and Saxena, 2009). In the present study, considerable proline accumulated under salt stress, while the plants with SA exhibited an increase compared with non-SA-treated ones. These results are partially similar to Misra and Saxena (2009), indicating that proline accumulation may contribute to osmotic adjustment at the cellular level, protect membrane integrity and consequently, mediate the damage induced by salt.

Studies have shown that the serious damage caused by salinity stress is partially due to the generation of reactive oxygen species (ROS), such as hydrogen peroxide (H_2_O_2_) and superoxide (${\text{O}}_{2}^{.-}$) (Asada, 2006). Our study agrees with this conclusion: salt stress increased the ${\text{O}}_{2}^{.-}$ production rate and H_2_O_2_ content by 56.5% (*P* < 0.05) and 60.0% (*P* < 0.05) under the 0.3% treatment, 111.4% (*P* < 0.05) and 97.5% (*P* < 0.05) under the 0.6% treatment, and 203.1% (*P* < 0.05) and 130.0% (*P* < 0.05), under the 0.9% treatment, respectively. However, this increase in ROS was reduced in plants that were treated with SA, compared with the non-SA-treated plants, which suggesting that SA could protect cells and sub-cellular systems from these ROS cytotoxic effects. In fact, plants can protect their tissues from the toxic effects of salt-accumulated ROS by using enzymes such as superoxide dismutase (SOD), catalase (CAT) and peroxidase (POD) (Verhagen et al., 2004). In the present study, SA induced the activities of these antioxidant enzymes in the *D. superbus* under the salinity condition (Figure 5). Other reports support this observation, i.e., that the SA-induced growth increase could be related to the enhanced activity of antioxidants, which protect the plants from oxidative damage (e.g., Hayat et al., 2008). Recently, studies have indicated that SA plays a major role in modulating antioxidant enzyme activities under salt stress, which allows a greater ability to withstand salinity-induced injury (Horvath et al., 2007; Harfouche et al., 2008).

Both carbon dioxide uptake and water vapor diffusion take place through the stomata. Slack (1974) examined stomatal density and distribution on the leaves of four apple varieties and found that stomatal development appeared to be related to environmental conditions, and its density apparently affected photosynthesis. In the current study, a progressive decrease in stomatal density and the stomata aperture was observed under salt stress (Figure 6, Table S3). The results are similar to those presented by Moyo et al. (2012), indicating that abnormal stomatal development and morphology were caused by salinity stress, which led to reduced functionality of the stomata and consequently, lower photosynthesis of the *D. superbus*. Compared with the non-SA-treated plants, the SA treatment increased the stomatal density, indicating that SA had a positive effect on stomatal development and morphology, which improved photosynthesis.

Photosynthesis only occurs in the chloroplasts, and the photoreaction is localized in the internal chloroplast membrane (i.e., the thylakoid). The current results showed that the chloroplasts became oblate or spheroidal, and the grana gradually became irregularly shaped with reduced lamellae and a disorderly thylakoid membrane structure with increasing salinity stress levels (Figure 7, Table S4). Even osmiophilic globules showed a slight increase in salt-stressed *D. superbus*. Lichtenthaler (2013) reported that osmiophilic globules were plastoglobuli in chloroplasts and were associated with membrane turnover/breakdown, senescence and also stress. These results in osmiophilic globules are similar to those of previous studies (Tang et al., 2015), indicating that salinity might cause abundant ROS (H_2_O_2_, ${\text{O}}_{2}^{.-}$) production and impair the thylakoids, subsequently decreasing photosynthesis and plant growth. Nevertheless, the SA-treated seedlings exhibited some well-developed grana and contained more thylakoids compared with non-treated plants, indicating that SA exerted a significantly positive effect on physiological function under salt stress, by alleviating the damage caused to the chloroplasts and consequently, promoting the resistance of *D. superbus* under salt stress.

In conclusion, the present study demonstrated that *D. superbus* is a salt-tolerant herbaceous flower, but severe salt stress can lead to growth inhibition and reduced photosynthesis, in addition to poor development of the photosynthetic apparatus and the accumulation of ROS. However, SA minimized the deleterious effect of salt on the growth and adaptation of *D. superbus*, which was attributed to the well-developed chloroplasts and high activity of the antioxidant enzymes. This is the first report on the salt tolerance mechanisms in *D. superbus*. The results of this study can make a positive contribution to the cultivation and promotion of other herbaceous flowers.

## Author contributions

XM and JZ: Design and do experiment and analyze data and complete the manuscript. XZ and RQ: Help to do experiment. QH: Amend manuscript.

### Conflict of interest statement

The authors declare that the research was conducted in the absence of any commercial or financial relationships that could be construed as a potential conflict of interest.

## Supplementary material

Table S1 **Fresh weight (FW), dry biomass, leaf mass ratio (LMR), and specific leaf weight (SLW) of D. superbus subjected to different levels of salt and salicylic acid**. T1, distilled water; T2, distilled water with 0.5 mmol SA; T3, 0.3% NaCl; T4, 0.3% NaCl with 0.5 mmol SA; T5, 0.6% NaCl; T6, 0.6% NaCl with 0.5 mmol SA; T7, 0.9% NaCl; T8, 0.9% NaCl with 0.5 mmol SA. The values presented are the means ± SE. Different letters indicate significant differences between treatments (*P* < 0.05); *n* = 5.

Table S2 **Effects of SA treatments on the net photosynthetic rate (Pn), transpiration rate (Tr), internal carbon dioxide concentration (Ci), and stomatal conductance (Gs) of *D. superbus* grown under salt stress**. T1, distilled water; T2, distilled water with 0.5 mmol SA; T3, 0.3% NaCl; T4, 0.3% NaCl with 0.5 mmol SA; T5, 0.6% NaCl; T6, 0.6% NaCl with 0.5 mmol SA; T7, 0.9% NaCl; T8, 0.9% NaCl with 0.5 mmol SA. Numbers followed by different letters indicate significant differences (*P* < 0.05) according to an LSD-test, *n* = 5.

Table S3 **Effects of SA treatments on the stomata density and stomatal aperture of *D. superbus* grown under salt stress**. T1, distilled water; T2, distilled water with 0.5 mmol SA; T3, 0.3% NaCl; T4, 0.3% NaCl with 0.5 mmol SA; T5, 0.6% NaCl; T6, 0.6% NaCl with 0.5 mmol SA; T7, 0.9% NaCl; T8, 0.9% NaCl with 0.5 mmol SA. Numbers followed by different letters indicate significant differences (*P* < 0.05) according to an LSD-test, *n* = 5.

Table S4 **Effects of SA treatments on the chloroplast ultrastructure of *D. superbus* grown under salt stress**. T1, distilled water; T2, distilled water with 0.5 mmol SA; T3, 0.3% NaCl; T4, 0.3% NaCl with 0.5 mmol SA; T5, 0.6% NaCl; T6, 0.6% NaCl with 0.5 mmol SA; T7, 0.9% NaCl; and T8, 0.9% NaCl with 0.5 mmol SA. Numbers followed by different letters indicate significant differences (*P* < 0.05) according to an LSD-test, *n* = 5.

## References

[B1] Arfan M. Athar H. R. Ashraf M. (2007). Does exogenous application of salicylic acid through the rooting medium modulate growth and photosynthetic capacity in two differently adapted spring wheat cultivars under salt stress? Plant Physiol. 6, 685–694. 10.1016/j.jplph.2006.05.010 16884826

[B2] Asada K. (2006). Production and scavenging of reactive oxygen species in chloroplasts and their functions. Plant Physiol. 141, 391–396. 10.1104/pp.106.082040 16760493 PMC1475469

[B3] Bittrich V. (1993). Caryophyllaceae, in Families and Genera of Flowering Plants, 2. vol. 2, Flowering Plants, Dicotyledons, eds Kubitzki K. Rohwer J. G. Bittrich V. (Berlin: Springer), 206–236.

[B4] Borsani O. Valpuesta V. Botella M. A. (2001). Evidence for a role of salicylic acid in the oxidative damage generated by NaCl and osmotic stress in Arabidopsis seedlings. Plant Physiol. 126, 1024–1034. 10.1104/pp.126.3.1024 11457953 PMC116459

[B5] Cao Y. Zhang Z. W. Xue L. W. Du J. B. Shang J. Xu F. Yuan S.. (2009). Lack of salicylic acid in Arabidopsis protects plants against moderate salt stress. Z. Naturforschung C 64, 231–238. 10.1515/znc-2009-3-414 19526718

[B6] Deng Y. Sha Q. Li C. Ye X. Tang R. (2012). Differential responses of double petal and multi petal jasmine to shading: II. Morphology, anatomy and physiology. Sci. Hortic. 144, 19–28. 10.1016/j.scienta.2012.06.031 22562019

[B7] Deshmukh P. S. Sairam R. K. Shukla D. S. (1991). Measurement of ion leakage as a screening technique for drought resistance in wheat genotypes. Indian J. Plant Physiol. 35, 89–911.

[B8] Díaz-Vivancos P. Clemente-Moreno M. J. Rubio M. Olmos E. García J. A. Martínez-Gómez P.. (2008). Alteration in the chloroplastic metabolism leads to ROS accumulation in pea plants in response to plum pox virus. J. Exp. Bot. 59, 2147–2160. 10.1093/jxb/ern082 18535298 PMC2413280

[B9] Dubey R. S. (2005). Photosynthesis in plants under stressful conditions, in Hand Book of Photosynthesis, 2nd Edn, ed Pessarakli M. (New York, NY: CRC Press; Taylor and Francis Group), 717–737.

[B10] Durner J. Klessig D. F. (1996). Salicylic acid is a modulator of tobacco and mammalian catalases. Plant Mol. Biol. 28, 28492–28501. 10.1074/jbc.271.45.28492 8910477

[B11] Fidalgo F. Santos A. Santos I. Salema R. (2004). Effects of long-term salt stress on antioxidant defence systems, leaf water relations and chloroplast ultrastructure of potato plants. Ann. Appl. Biol. 145, 185–192. 10.1111/j.1744-7348.2004.tb00374.x

[B12] Giannopotitis C. N. Ries S. K. (1977). Superoxide dismutase in higher plants. Plant Physiol. 59, 309–314. 10.1104/pp.59.2.309 16659839 PMC542387

[B13] Gossett D. R. Millhollon E. P. Lucas M. C. (1994). Antioxidant response to NaCI Stress in salt-tolerant and salt-sensitive cultivars of cotton. Crop Sci. 34, 706–714. 10.2135/cropsci1994.0011183X003400030020x

[B14] Guidi L. Tonini M. Soldatini G. F. (2000). Effects of high light and ozone fumigation on photosynthesis in *Phaseolus vulgaris*. Plant Physiol. Biochem. 38, 717–725. 10.1016/S0981-9428(00)01172-4

[B15] Gunes A. Inal A. Alpaslan M. Eraslan F. Bagci E. G. Cicek N. (2007). Salicylic acid induced changes on some physiological parameters symptomatic for oxidative stress and mineral nutrition in maize (*Zea mays* L.) grown under salinity. J. Plant Physiol. 164, 728–736. 10.1016/j.jplph.2005.12.009 16690163

[B16] Harfouche A. L. Rugini E. Mencarelli F. Botondi R. Muleo R. (2008).Salicylic acid induces H_2_O_2_ production and endochitinase gene expression but not ethylene biosynthesis in *Castanea sativa in vitro* model system. Plant Physiol. 165, 734–744. 10.1016/j.jplph.2007.03.010 17765360

[B17] Hayat Q. Hayat S. Irfan M. Ahmad A. (2010). Effect of exogenous salicylic acid under changing environment: a review. Environ. Exp. Bot. 68, 14–25. 10.1016/j.envexpbot.2009.08.005

[B18] Hayat S. Hasan S. A. Fariduddin Q. Ahmad A. (2008). Growth of tomato (Lycopersicon esculentum) in response to salicylic acid under water stress. Plant Interact. 3, 297–304. 10.1080/17429140802320797

[B19] Hiromasa U. Noboru K. (2006). The germination traits of gynodioecious *Dianthus superbus* L. var. longicalycinus (Maxim.) Williams. J. Jpn. Soc. Revegetation Technol. 32, 122–126. 10.7211/jjsrt.32.122

[B20] Hooftman D. A. P. van Kleunen M. Diemer M. (2003). Effects of habitat fragmentation on the fitness of two common wetland species, Carex davalliana and Succisa pra-tensis. Oecologia 134, 350–359. 10.1007/s00442-002-1096-0 12647142

[B21] Horvath E. Szalai G. Janda T. (2007). Induction of abiotic stress tolerance by salicylic acid signaling. Plant Growth Regul. 26, 290–300. 10.1007/s00344-007-9017-4

[B22] Iqbal M. Ashraf M. Jamil A. Ur-Rehman S. (2006). Does seed priming induce changes in the levels of some endogenous plant hormones in hexaploid wheat plants under salt stress?. J. Integr. Plant Biol. 48, 181–189. 10.1111/j.1744-7909.2006.00181.x

[B23] Kováčik J. Grúz J. Bačkor M. Strnad M. Repčák M. (2009). Salicylic acid-induced changes to growth and phenolic metabolism in *Matricaria chamomilla* plants. Plant Cell Rep. 28, 135–143. 10.1007/s00299-008-0627-5 18972114

[B24] Kinga K. (2014). The life-history traits and seedling recruitment of *Dianthus superbus* L. in different stages of meadow overgrowing. Pol. Bot. Soc. 67, 23–30. 10.5586/aa.2014.022

[B25] Klessig D. F. Malamy J. (1994). The salicylic acid signal in plants. Plant Mol. Biol. 26, 1439–1458. 10.1007/BF00016484 7858199

[B26] Li T. Hu Y. Y. Du X. H. Tang H. Shen C. H. Wu J. S.. (2014). Salicylic acid alleviates the adverse effects of salt stress in *Torreya grandis* cv. merrillii seedlings by activating photosynthesis and enhancing antioxidant systems. PLoS ONE 9:e109492. 10.1371/journal.pone.0109492 25302987 PMC4193794

[B27] Lichtenthaler H. K. (1987). [34] Chlorophylls and carotenoids: pigments of photosynthetic biomembranes. Methods Enzymol. 148, 350–382. 10.1016/0076-6879(87)48036-1

[B28] Lichtenthaler H. K. (2013). Plastoglobuli, thylakoids, chloroplast structure and development of plastids, in Plastid Development in Leaves during Growth and Senescence, eds Biswal B. Krupinska K. Biswal U. C. (Dordrecht: Springer), 337–361.

[B29] Lutts S. Kinet J. M. Bouharmont J. (1995). Changes in plant response to NaCl during development of rice (*Oryza sativa* L.) varieties differing in salinity resistance. J. Exp. Bot. 46, 1843–1852. 10.1093/jxb/46.12.1843

[B30] Ma X. Song L. Yu W. Hu Y. Liu Y. Wu J.. (2015). Growth, physiological, and biochemical responses of *Camptotheca acuminata* seedlings to different light environments. Front. Plant Sci. 6:321. 10.3389/fpls.2015.00321 26005446 PMC4424855

[B31] Maliakal S. K. McDonnell K. Dudley S. A. Schmitt J. (1999). Effects of red to far-red ratio and plant density on biomass allocation and gas exchange in *Impatiens capensis*. Int. J. Plant Sci. 160, 723–733. 10.1086/314157

[B32] Misra N. Saxena P. (2009). Effect of salicylic acid on proline metabolism in lentil grown under salinity stress. Plant Sci. 177, 181–189. 10.1016/j.plantsci.2009.05.007

[B33] Molina A. Bueno P. Marin M. C. Rodrıiguez-Rosales M. P. Belver A. Venema K. (2002). Involvement of endogenous salicylic acid content, lipoxygenase and antioxidant enzyme activities in the response of tomato cell suspension cultures to NaCl. New Phytol. 156, 409–415. 10.1046/j.1469-8137.2002.00527.x 33873571

[B34] Moyo M. Finnie J. F. Van Staden J. (2012). Microculture effects on leaf epidermis and root structure in *Sclerocarya birrea* subsp. caffra. South Afr. J. Bot. 78, 170–177. 10.1016/j.sajb.2011.06.011

[B35] Munns R. (2005). Genes and salt tolerance: bringing them together. New Phytol 167, 645–663. 10.1111/j.1469-8137.2005.01487.x 16101905

[B36] Opdekamp W. Beauchard O. Backx H. Fran-ken F. Cox T. J. S. van Diggelen R. (2012). Effects of mowing cessation and hydrology on plant trait distribution in natural fen meadows. Acta Oecologica 39, 117–127. 10.1016/j.actao.2012.01.011

[B37] Parida A. K. Das A. B. (2005). Salt tolerance and salinity effects on plants: a review. Ecotoxicol. Environ. Saf. 60, 324–349. 10.1016/j.ecoenv.2004.06.010 15590011

[B38] Patterson B. D. MacRae E. A. Ferguson I. B. (1984). Estimation of hydrogen peroxide in plant extracts using titanium (IV). Anal. Biochem. 139, 487–492. 10.1016/0003-2697(84)90039-3 6476384

[B39] Rosenthal G. (2010). Secondary succession in a fallow central European wet grassland. Flora 205, 153–160. 10.1016/j.flora.2009.02.003

[B40] Roussos P. A. Gasparatos D. Tsantili E. Pontikis C. A. (2007). Mineral nutrition of jojoba explants *in vitro* under sodium chloride salinity. Sci. Hortic. 114, 59–66. 10.1016/j.scienta.2007.05.001

[B41] Roussos P. Gasparatos D. Kyriakou C. Tsichli K. Tsantili E. Haidouti C. (2013). Growth, nutrient status and biochemical changes in sour orange (*Citrus aurantium* L.) plants subjected to sodium chloride stress. Commun. Soil Sci. Plant Anal. 44, 805–816. 10.1080/00103624.2013.749438

[B42] Senaratna T. Touchell D. Bunn E. Dixon K. (2000). Acetyl salicylic acid (aspirin) and salicylic acid induce multiple stress tolerance in bean and tomato plants. Plant Growth Regul. 30, 157–161. 10.1023/A:1006386800974

[B43] Shakirova F. M. Sakhabutdinova A. R. Bezrukova M. V. Fatkhutdinova R. A. Fatkhutdinova D. R. (2003). Changes in the hormonal status of wheat seedlings induced by salicylic acid and salinity. Plant Sci. 164, 317–322. 10.1016/S0168-9452(02)00415-6

[B44] Shao Q. S. Wang H. Z. Guo H. P. Zhou A. C. Huang Y. Q. Sun Y. L.. (2014). Effects of shade treatments on photosynthetic characteristics, chloroplast ultrastructure, and physiology of Anoectochilus roxburghii. PLoS ONE 9:e85996. 10.1371/journal.pone.0085996 24516523 PMC3917826

[B45] Shen C. Hu Y. Du X. Li T. Tang H. Wu J. (2014). Salicylic acid induces physiological and biochemical changes in *Torreya grandis* cv. Merrillii seedlings under drought stress. Trees 28, 961–970. 10.1007/s00468-014-1009-y

[B46] Shim I. S. Momose Y. Yamamoto A. Kim D. W. Usui K. (2009). Inhibition of catalase activity by oxidative stress and its relationship to salicylic acid accumulation in plants. Plant Growth Regul. 39, 285–292. 10.1023/A:1022861312375

[B47] Slack E. M. (1974). Studies of stomatal distribution on the leaves of four apple varieties. J. Hortic. Sci. 49, 95–103. 10.1080/00221589.1974.11514555

[B48] Snider J. L. Choinski J. S. Wise R. R. (2009). Juvenile Rhus glabra leaves have higher temperatures and lower gas exchange rates than mature leaves when compared in the field during periods of high irradiance. J. Plant Physiol. 166, 686–696. 10.1016/j.jplph.2008.09.007 18849091

[B49] Szalai G. Tari I. Janda T. Pestena'cz A. Pa'ldi E. (2000). Effects of cold acclimation and salicylic acid on changes in ACC and MACC contents in maize during chilling. Biol. Plant 43, 637–640. 10.1023/A:1002824721597

[B50] Takemura T. Hanagata N. Sugihara K. Babab S. Karubea I. Dubinskya Z. (2000). Physiological and biochemical responses to salt stress in the mangrove, *Bruguiera gymnorrhiza*. Aquat. Bot. 68, 15–28. 10.1016/S0304-3770(00)00106-6

[B51] Tang H. Hu Y. Y. Yu W. W. Song L. L. Wu J. S. (2015). Growth, photosynthetic and physiological responses of *Torreya grandis* seedlings to varied light environments. Trees 29, 1011–1022. 10.1007/s00468-015-1180-9

[B52] Thomas R. L. Jen J. J. Morr C. V. (1982). Changes in soluble and bound peroxidase-IAA oxidase during tomato fruit development. J. Food Sci. 47, 158–161. 10.1111/j.1365-2621.1982.tb11048.x

[B53] Verhagen J. Put M. Zaal F. van Keulen H. (2004). Climate Change and Drought Risks for Agriculture. The Impact of Climate Change on Drylands. Dordrecht: Springer; Kluwer Academic Publishers 49–59.

[B54] Wang A. G. Luo G. H. (1990). Quantitative relation between the reaction of hydroxylamine and superoxide anion radicals in plants. Plant Physiol. Commun. 6, 55–57.

[B55] Yalpani N. Enyedi A. J. Leo J. Raskin I. (1994). Ultraviolet light and ozone stimulate accumulation of salicylic acid and pathogenesis related proteins and virus resis-tance in tobacco. Planta 193, 373–376. 10.1007/BF00201815

