# Supplementary material for: Salicylic Acid Alleviates the Adverse Effects of Salt Stress on Dianthus superbus (Caryophyllaceae) by Activating Photosynthesis, Protecting Morphological Structure, and Enhancing the Antioxidant System
Source: Front Plant Sci. 2017 Apr 21;8:600. doi: 10.3389/fpls.2017.00600 (PMC5399920; doi:10.3389/fpls.2017.00600)
Supplement: Table S1 — Fresh weight (FW), dry biomass, leaf mass ratio (LMR), and specific leaf weight (SLW) of D. superbus subjected to different levels of salt and salicylic acid. T1, distilled water; T2, distilled water with 0.5 mmol SA; T3, 0.3% NaCl; T4, 0.3% NaCl with 0.5 mmol SA; T5, 0.6% NaCl; T6, 0.6% NaCl with 0.5 mmol SA; T7, 0.9% NaCl; T8, 0.9% NaCl with 0.5 mmol SA. The values presented are the means ± SE. Different letters indicate significant differences between treatments (P < 0.05); n = 5. [file Table1.DOC]

| Treatment | Fresh weight (g) | Dry Biomass  (g) | leaf mass  ratio | specific leaf weight |
| --- | --- | --- | --- | --- |
| T1 | 47.30 ±2.36ab | 19.70 ± 0.96bc | 0.26 ± 0.02d | 0.029 ± 0.002d |
| T2 | 50.19 ± 2.24a | 21.37 ± 0.21a | 0.28 ± 0.03cd | 0.028 ± 0.002d |
| T3 | 42.24 ± 1.49c | 18.97 ± 1.16c | 0.38 ± 0.01bc | 0.032 ± 0.003d |
| T4 | 49.52 ± 3.44a | 20.54 ± 0.97ab | 0.40 ± 0.03b | 0.029 ± 0.002d |
| T5 | 33.99 ± 2.17d | 16.24 ± 0.13d | 0.32 ± 0.14c | 0.036 ± 0.002c |
| T6 | 45.55 ± 1.80c | 18.33 ± 0.28c | 0.53 ± 0.03a | 0.041 ± 0.002b |
| T7 | 29.05 ± 1.38e | 14.76 ± 0.90e | 0.28 ± 0.07cd | 0.045 ± 0.003a |
| T8 | 27.73 ± 0.82e | 11.83 ± 1.15f | 0.41 ± 0.03b | 0.043 ± 0.003ab |

Table 1
