# Supplementary material for: Salicylic Acid Alleviates the Adverse Effects of Salt Stress on Dianthus superbus (Caryophyllaceae) by Activating Photosynthesis, Protecting Morphological Structure, and Enhancing the Antioxidant System
Source: Front Plant Sci. 2017 Apr 21;8:600. doi: 10.3389/fpls.2017.00600 (PMC5399920; doi:10.3389/fpls.2017.00600)
Supplement: Table S2 — Effects of SA treatments on the net photosynthetic rate (Pn), transpiration rate (Tr), internal carbon dioxide concentration (Ci), and stomatal conductance (Gs) of D. superbus grown under salt stress. T1, distilled water; T2, distilled water with 0.5 mmol SA; T3, 0.3% NaCl; T4, 0.3% NaCl with 0.5 mmol SA; T5, 0.6% NaCl; T6, 0.6% NaCl with 0.5 mmol SA; T7, 0.9% NaCl; T8, 0.9% NaCl with 0.5 mmol SA. Numbers followed by different letters indicate significant differences (P < 0.05) according to an LSD-test, n = 5. [file Table2.DOC]

| Treatments | Pn  (μmol·m^-2^·s^-1^) | Ci  (μmol·mol^-1^) | Tr  (μmol·H_2_O·m^-2^·s^-1^) | Gs  (mmol·m^-2^·s^-1^) |
| --- | --- | --- | --- | --- |
| T1 | 4.32 ± 0.23ab | 253.1 ± 13.4b | 2.87 ± 0.38a | 0.31 ± 0.002a |
| T2 | 4.50 ± 0.18a | 255.6 ± 12.7b | 2.82 ± 0.46ab | 0.32 ± 0.016a |
| T3 | 3.88 ± 0.15c | 228.4 ± 18.7c | 2.46 ± 0.58c | 0.24 ± 0.001b |
| T4 | 4.23 ± 0.13b | 248.3 ± 12.8b | 2.71 ± 0.23b | 0.27 ± 0.001ab |
| T5 | 3.32 ± 0.21d | 217.2 ± 21.1d | 2.27 ± 0.35e | 0.21 ± 0.001c |
| T6 | 3.61 ± 0.13cd | 224.8 ± 14.7c | 2.38 ± 0.18d | 0.22 ± 0.002c |
| T7 | 3.02 ± 0.24e | 272.6 ± 17.5a | 2.13 ± 0.51f | 0.21 ± 0.001c |
| T8 | 2.93 ± 0.19e | 270.3 ± 16.3a | 2.11 ± 0.45f | 0.20 ± 0.001c |

Table 2
