# Supplementary material for: Salicylic Acid Alleviates the Adverse Effects of Salt Stress on Dianthus superbus (Caryophyllaceae) by Activating Photosynthesis, Protecting Morphological Structure, and Enhancing the Antioxidant System
Source: Front Plant Sci. 2017 Apr 21;8:600. doi: 10.3389/fpls.2017.00600 (PMC5399920; doi:10.3389/fpls.2017.00600)
Supplement: Table S3 — Effects of SA treatments on the stomata density and stomatal aperture of D. superbus grown under salt stress. T1, distilled water; T2, distilled water with 0.5 mmol SA; T3, 0.3% NaCl; T4, 0.3% NaCl with 0.5 mmol SA; T5, 0.6% NaCl; T6, 0.6% NaCl with 0.5 mmol SA; T7, 0.9% NaCl; T8, 0.9% NaCl with 0.5 mmol SA. Numbers followed by different letters indicate significant differences (P < 0.05) according to an LSD-test, n = 5. [file Table3.DOC]

Table 3

| Treatments | Stomatal aperture (μm) | Stomatal density (mm^-2^) |
| --- | --- | --- |
| T1 | 5.09 ± 0.20a | 525.39 ± 13.36a |
| T2 | 5.14 ± 0.32a | 516.21 ± 12.25a |
| T3 | 2.59 ± 0.09c | 416.21 ± 6.62c |
| T4 | 3.23 ± 0.21b | 498.61 ± 6.52b |
| T5 | 2.03 ± 0.07d | 346.98 ± 8.72e |
| T6 | 2.42 ± 0.07c | 387.29 ± 4.78d |
| T7 | 1.60 ± 0.02e | 312.42 ± 3.87f |
| T8 | 1.69 ± 0.07e | 314.73 ± 7.41f |
