# Supplementary material for: Salicylic Acid Alleviates the Adverse Effects of Salt Stress on Dianthus superbus (Caryophyllaceae) by Activating Photosynthesis, Protecting Morphological Structure, and Enhancing the Antioxidant System
Source: Front Plant Sci. 2017 Apr 21;8:600. doi: 10.3389/fpls.2017.00600 (PMC5399920; doi:10.3389/fpls.2017.00600)
Supplement: Table S4 — Effects of SA treatments on the chloroplast ultrastructure of D. superbus grown under salt stress. T1, distilled water; T2, distilled water with 0.5 mmol SA; T3, 0.3% NaCl; T4, 0.3% NaCl with 0.5 mmol SA; T5, 0.6% NaCl; T6, 0.6% NaCl with 0.5 mmol SA; T7, 0.9% NaCl; and T8, 0.9% NaCl with 0.5 mmol SA. Numbers followed by different letters indicate significant differences (P < 0.05) according to an LSD-test, n = 5. [file Table4.DOC]

| Treatments | Chloroplast length (μm) | Chloroplast width(μm) | Grana number | Grana thickness (μm) | Number of grana lamellae | Number of osmiophilic gra-nule |
| --- | --- | --- | --- | --- | --- | --- |
| T1 | 4.78 ± 0.29a | 1.77 ± 0.19e | 14 ± 1.0a | 0.39 ± 0.06a | 15 ± 1.0a | 3.36 ± 1.52e |
| T2 | 4.68 ± 0.30a | 1.82 ± 0.03e | 13.7 ± 1.15a | 0.35 ± 0.07ab | 14.33 ± 1.15ab | 4.01 ± 1.73e |
| T3 | 4.13 ± 0.07c | 2.4 ± 0.23cd | 9.33 ± 1.53c | 0.21 ± 0.03c | 11.39 ± 0.54c | 8.66 ± 0.58c |
| T4 | 4.59 ± 0.31ab | 2.19 ± 0.04d | 12 ± 1.03b | 0.31 ± 0.06b | 13.33 ± 0.57b | 6.33± 0.58d |
| T5 | 4.09 ± 0.12c | 2.94 ± 0.07b | 8.66 ± 0.58cd | 0.15 ± 0.01cd | 9.43± 0.45d | 11.33 ± 1.52b |
| T6 | 4.28 ± 0.22bc | 2.52 ± 0.17c | 10.1 ± 0.12c | 0.20 ± 0.02c | 10.96 ± 0.07c | 8.32± 0.56c |
| T7 | 3.97 ± 0.04c | 3.76 ± 0.07a | 7.33 ± 0.58d | 0.11 ± 0.01d | 8.14 ± 0.24e | 14 ± 1.0a |
| T8 | 4.17 ± 0.16c | 3.05 ± 0.25a | 7 ± 0.95d | 0.10 ± 0.02d | 7.66 ± 0.57e | 13.67 ± 0.57a |

Table 4
